# Electrodeposition of ZnO window layer for an all-atmospheric fabrication process of chalcogenide solar cell

**DOI:** 10.1038/srep08961

**Published:** 2015-03-10

**Authors:** Fabien Tsin, Amélie Venerosy, Julien Vidal, Stéphane Collin, Johnny Clatot, Laurent Lombez, Myriam Paire, Stephan Borensztajn, Cédric Broussillou, Pierre Philippe Grand, Salvador Jaime, Daniel Lincot, Jean Rousset

**Affiliations:** 1 EDF R&D, 6 quai Watier, 78400 Chatou Cedex, France; 2 CNRS, 6 quai Watier, 78400 Chatou Cedex, France; 3 IRDEP, Institute of Research and Development on Photovoltaic Energy, UMR 7174 CNRS EDF Chimie ParisTech, 6 quai Watier, 78400 Chatou Cedex, France; 4 LPN, Laboratoire for Photonics and Nanostructures, UPR 20 CNRS, Route de Nozay, 91460 Marcoussis, France; 5 NEXCIS Photovoltaic Technology, 13790 Rousset, France

## Abstract

This paper presents the low cost electrodeposition of a transparent and conductive chlorine doped ZnO layer with performances comparable to that produced by standard vacuum processes. First, an in-depth study of the defect physics by ab-initio calculation shows that chlorine is one of the best candidates to dope the ZnO. This result is experimentally confirmed by a complete optical analysis of the ZnO layer deposited in a chloride rich solution. We demonstrate that high doping levels (>10^20^ cm^−3^) and mobilities (up to 20 cm^2^ V^−1^ s^−1^) can be reached by insertion of chlorine in the lattice. The process developed in this study has been applied on a CdS/Cu(In,Ga)(Se,S)_2_ p-n junction produced in a pilot line by a non vacuum process, to be tested as solar cell front contact deposition method. As a result efficiency of 14.3% has been reached opening the way of atmospheric production of Cu(In,Ga)(Se,S)_2_ solar cell.

Transparent conductive oxides (TCO) are used in a wide range of optoelectronic devices such as light emitting diodes, chemical sensors, touchscreens or in photovoltaics^1^. Among the possible large band gap compounds, ZnO received much attention because it combines low cost components and tunability of its optoelectronic properties^2^. At industrial level, ZnO compounds are already used in the fabrication lines of various photovoltaic modules, especially those of the Cu(In,Ga)(Se,S)_2_ (referred as CIGS) based solar cell^3^. In most cases, the deposition of the ZnO window layer on large surface is ensured by vacuum processes which demand massive investments and high level of operating expenses. Indeed, according to cost studies^4^, the deposition of the ZnO front contact (assuming MOCVD process) corresponds to 13% of the total cost of the module fabrication, ranking second most expensive material deposition step after the one of the CIGS absorber (by coevaporation or sputtering/annealing). Our research focuses on the development of a low cost process for the production of high quality ZnO layer on large surfaces. The electrodeposition method is a very interesting candidate for this task. This atmospheric technique takes place in water solution and at atmospheric pressure using low-cost and non toxic precursors, and is known to be easily up scalable. The electrodeposition mechanism of ZnO was elucidated nearly 20 years ago^5^,^6^, thanks to studies on the influence of the different growth parameters. As a consequence, different bath formulations have been proposed and efficient methods allow the production of dense layers of ZnO with high crystallinity and high transparency [For example Ref. 7]. But surprisingly, only few papers focus on the electronic properties and in particular on the doping level of the electrodeposited material^8^,^9^,^10^,^11^ and even fewer dealt with electrodeposited ZnO films as CIGS solar cell front contact^12^,^13^,^14^. One of the reasons is the sensitivity of ZnO to the pH conditions, as this reduces the choice of possible doping elements. For example, the commonly used doping agent aluminum is quasi insoluble at the pH needed for the electrodeposition process (close to neutral). We have demonstrated an innovative way to overpass this limitation by using chlorine as the doping element^9^. High free carrier concentrations (>10^20^ cm^−3^) have been reached by introducing chloride ions into the electrochemical bath. However, the fine characterization of the electrical and optical properties of the films was particularly challenging due to the presence of a conductive substrate required by the electrodeposition process.

Herein, we report a comprehensive study on the production of high quality transparent conductive oxide by electrodeposition, from theoretical calculations to device fabrication. First, we evaluated chlorine as a doping element by ab initio calculations. Then we confronted it to the experimental data. To acquire the TCO experimental optoelectronic properties, a lift off method of the ZnO layer from the substrate was developed, and allowed a full optical analysis to determine the doping level and intragrain mobility of the electrodeposited ZnO layer. The influence of the chloride concentration in the bath and thermal post treatment on the optoelectronic properties of the ZnO film has been explored.

Finally, those layers were tested in actual solar devices in close collaboration with the company NEXCIS. This latter develops non vacuum two step process of CIGS deposition on large surface consisting in an electrodeposition of a precursor layer followed by a thermal treatment. This company recently claims the fabrication of a certified 60 × 120 cm^2^ module at 13.7% aperture efficiency. Our goal was to adapt the ZnO electrodeposition to the Mo/CIGS/CdS substrate produced in the NEXCIS pilot line. The sputtered i-ZnO/ZnO:Al bi layer classically used in the CIGS industrial process was substituted by a single Cl doped ZnO (referred as ZnO:Cl) directly deposited on the CdS surface without any pretreatment. The efficiencies obtained with this new window layer, up to 14.3% (on 0.5 cm^2^ cells) are in the same range to those measured with the sputtered references on the same absorber. At the best of the author's knowledge such high efficiency is reported for the first time for a CIGS based cell made by successive simple atmospheric growth processes. These very encouraging results allow considering the possibility to produce CIGS solar cell with an atmospheric production line (except for the sputtered back contact).

## Simulation of the doping of the ZnO by chlorine introduction

**Figure 1:**
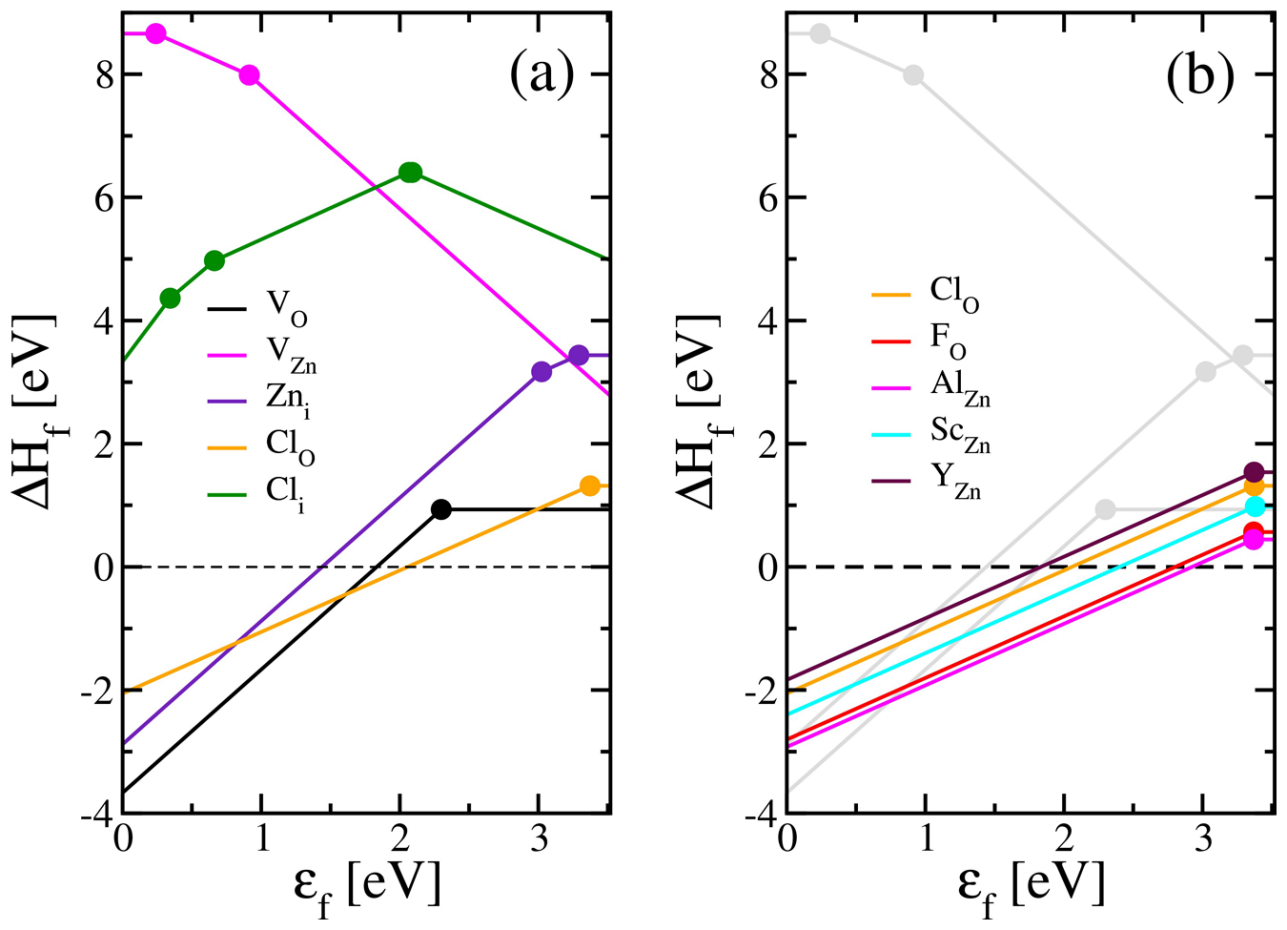
Defect formation enthalpies in ZnO by ab-initio calculations. Defect formation enthalpies of (a) intrinsic defects and Cl-related defects and (b) extrinsic dopants with respect to Fermi level ε_f_ under O-rich growth condition.

Ab initio calculations (theoretical details are given in the supplementary materials) allow for a deeper insight on the defect physics of Cl-doped ZnO (refered as ZnO:Cl). Figure 1a displays the formation enthalpies of Cl-related defects together with the ones of intrinsic defects of ZnO.

According to total energy calculations, Cl atom prefers the antisite position on oxygen (referred as Cl_O_) rather than the interstitial one (referred as Cl_i_). Such defect configuration could be inferred from the crystal structure of zinc chloride ZnCl_2_, which crystallizes in i-42d space groupe and adopts a local tetrahedral coordination. Zn-Cl bond length in ZnCl_2_ is calculated at 2.27 Å while Zn-Cl bond lengths in ZnO:Cl range between 2.33 and 2.41 Å depending on the charge state. Cl_O_ antisite displays a rather shallow transition level ε(0/+) close to the conduction edge similar to the case of Al_Zn_ antisites. As a consequence, such defects can be considered as an efficient n-type dopant. On the other hand, Cl interstitials have large formation enthalpies with an ambipolar character, *i.e.* stabilizing a handful of charge states depending of the position of the Fermi level. For Fermi level close to the conduction band minimum, the state charge -1 has the lowest formation enthalpy, meaning that Cl interstitials act as compensating defect with respect to n-type doping. However, its rather large formation energy indicates that only low concentration of such interstitial sites will be occupied by Cl atoms.

The n-type doping capability of a material is limited by (i) the formation of electron killer (such as Zn vacancies or Chlorine interstitial), (ii) the formation of competing phases limiting incorporation of dopant atoms and (iii) the size of the dopant ion. Condition (ii) can be usually relaxed when dealing with thin films grown under non equilibrium conditions. It is also noticed that condition (i) is never satisfied in ZnO as Zn vacancies have significantly high formation enthalpy under either O-rich or O-poor growth condition. If one considers cation doping in ZnO, Al is the best dopant because the associated competing phase *i.e* Al_2_O_3_ is 0.5 eV/atom less stable than competing phases associated to Sc and Y, namely Sc_2_O_3_ and Y_2_O_3_. Nevertheless, ionic radius of Al is considerably smaller than the one of Zn that it replaces when forming Al-on-Zn antisite *i.e.* r_Al_ = 67.5 ppm and r_Zn_ = 88.5 ppm. On the other hand, Sc ionic radius is surprisingly close to the one of Zn, r_Sc_ = 88 ppm. This translates into the defect formation enthalpies as, if one were not to consider competing phases, Sc-on-Zn antisite would have the lowest formation enthalpy among all extrinsic cationic dopants considered in this study. The same analysis can be carried out for anion doping. First, it is observed that ZnF_2_ has a formation enthalpy at room temperature 1.2 eV/atom lower than the one of ZnCl_2_, thus preventing F insertion into ZnO. Yet, formation enthalpy of F-on-O antisite is as low as the one of Al-on-Zn antisite owing to the close match of the ionic radii between F and O. On the other hand, Cl ionic radius is 30% larger than the one of O and such as in the case of Y where Y ionic radius is 18% larger than the one of Zn, the energy cost to form those antisite defects is significantly increased. In this study, Fluorine appears as one of the best candidate to dope the ZnO and high performances of ZnO:F have already been demonstrated^15^. But the toxicity of this element and its associated compounds tends to make it difficult to use at an industrial scale. Moreover, Cl-based doping presents several advantages in the case of an electrochemical process: chloride ions are non toxic, soluble in large amount in pH conditions (close to the neutrality) of the electrolyte and electrochemically inactive in the potential range in which the electrodeposition takes place.

The above analysis shows the potential of anion doping compared to cation doping for obtaining n-type doped ZnO. Cationic substitutional doping is somehow limited by the very low formation enthalpy of native oxides such as Al_2_O_3_, Sc_2_O_3_ or Y_2_O_3_ compared to the one of ZnO, requiring the use of non-equilibrium growth techniques to include significant amount of cationic dopant in ZnO. On the other hand, anionic substitutional doping is eased by the higher formation enthalpy of halide compounds compared to the binary oxides considered here.

## Morphology and composition aspects

**Figure 2:**
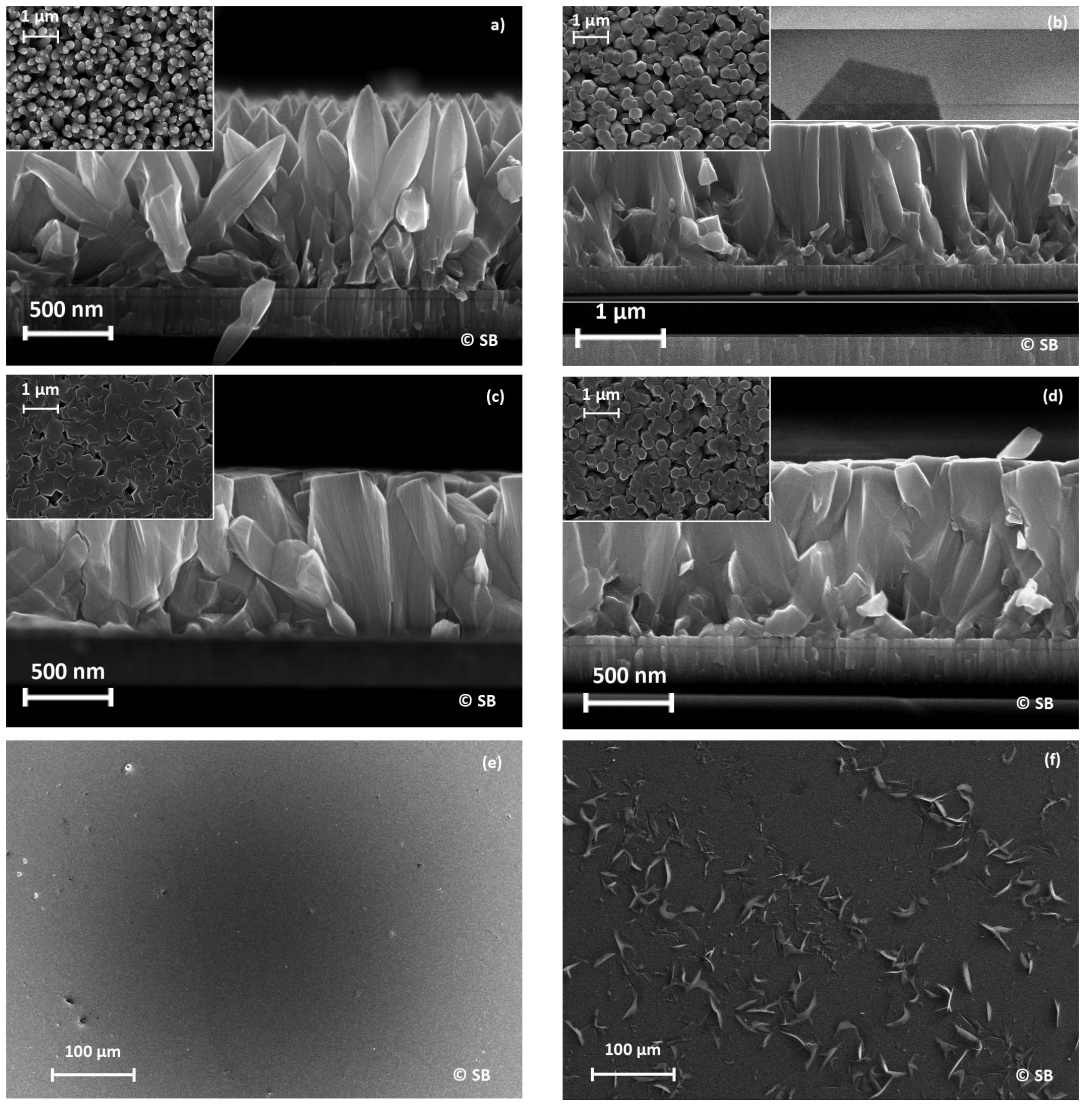
Morphology (surface and cross section) of ZnO layers deposited from chloride bath on glass/Mo substrate at 0.05 M (a), 0.1 M (b), 0.15 M (c, e) and 0.2 M (d, f).

The SEM images of ZnO thin films electrodeposited in electrolytes with different chloride contents are shown in Figure 2. The morphology of the deposited layer widely depends on the chloride concentration in the bath. At low Cl concentration, the ZnO layer is not compact mainly formed by pen-shaped columns (figure 2a). Such type of morphology usually induces high sheet resistance and is not suitable for the targeted front contact application. At Cl concentration above 0.1 M, the classical columnar hexagonal structure is obtained^5^. Moreover, the increase of the chloride concentration promotes the compacity of the zinc oxide layer leading to a broadening of the ZnO columns. Thus, the column diameter ranges from 200 nm at [Cl] = 0.05 M to 400 nm at [Cl] = 0.15 M. This phenomenon has already been reported by Tena-Zaera et al. for ZnO nanowires^16^,^17^. In this study, the broadening of ZnO nanowires was attributed to a c-axis blocking behavior of the chloride adsorption at the top of the column, promoting a lateral growth of the zinc oxide. For chlorine concentration superior or equal to 0.2 M, chlorine rich platelets are clearly visible on the low magnificence image (figure 2f) and could be identified as hydroxochloride compound. Indeed, the formation of Zn_5_(OH)_8_Cl_2_ has been reported in a previous study^18^ when the chloride and/or the zinc concentration is increased in the electrolyte. The appearance of this chlorine rich compound seems to limit the chloride effect on the ZnO growth leading to the decrease of the column diameter to 300 nm.

Energy dispersive spectrometry measurements show that the atomic chlorine content in the ZnO layer linearly increases as a function of the chloride ions concentration dissolved in the bath (see Figure 3 in supplementary materials). Indeed the Cl^−^ atomic percent ranges from 1.2% for [Cl] = 0.05 M to 1.75% for [Cl] = 0.175 M. The chlorine content measured at 0.2 M (at. % Cl = 1.7%) does not follow this trend. The formation of Cl-rich platelets at this concentration as seen in Fig. 2f seems to stop the introduction of chlorine in the ZnO lattice. This result is consistent with previously published results^19^,^20^ where the insertion of chloride ions in a ZnO thin film electrodeposited in chloride rich electrolyte ([Cl] = 0.1 M) has been investigated by means of X-ray photoelectron spectroscopy (XPS) and Secondary Ion Mass Spectrometry (SIMS): both the inclusion of Cl in ZnO films and uniform depth profile of the chloride ion concentration were then confirmed.

## Optical Properties

The optical properties of ZnO are deduced from the fit of the reflectance spectra with the Drude model. The theoretical background of this model can be found in the supplementary materials.

### Reference samples

**Figure 3:**
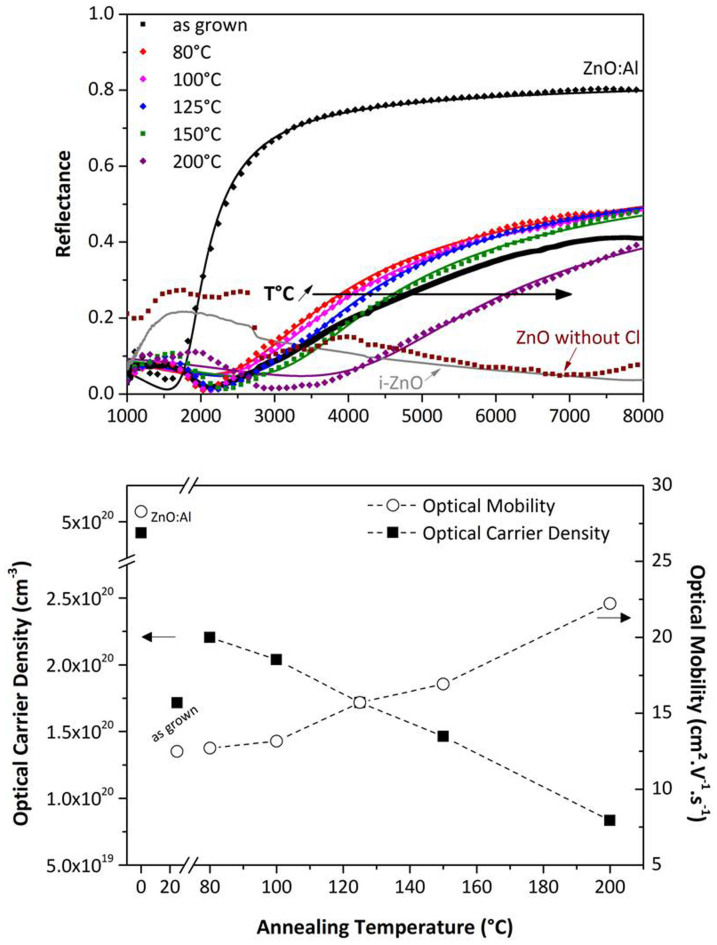
Impact of the post treatment temperature on the optoelectronic properties of the electrodeposited ZnO. Top: Experimental and simulated reflectance spectra of electrodeposited ZnO films after the lift-off step (the method is described in the supplementary method section), as a function of the annealing temperature compared to i-ZnO and ZnO:Al reference materials. Bottom: Optical mobility and carrier concentration extracted from the optical data.

In order to validate our approach the doping level and the mobility of the free carrier material are determined for the sputtered ZnO:Al and compared to those obtained by direct electrical means such as Hall effect measurement. This material shows a high reflectance, up to 80% (Figure 3), in infrared range which is the fingerprint of a high doping level. Thus, an optical carrier density of 5 × 10^20^ cm^−3^ and an optical mobility of 28 cm^2^.V^−1^.s^−1^ can be extracted from fit of the spectrum. The same doping level value is determined by electrical measurement, but the Hall mobility (μ_Hall_ = 23 cm^2^.V^−1^.s^−1^) is slightly lower than the optical one. Under NIR excitation the average electrons path length is much smaller than the typical grain size and is in the range of few nanometers. Thus, the difference between these two mobilities values can be attributed to grain boundaries which affect the long distance lateral transport of the electrons. Such effect remains weak because the high doping level enhances the transport through grain boundaries by tunneling effect^21^,^22^.

The spectra obtained for a chlorine-free ZnO film electrodeposited in a nitrate electrolyte is presented in Figure 3 and compared to that of an intrinsic sputtered material. Their shapes are similar: no reflectance signal is measured in the infrared range, as expected for a poorly doped material. On the contrary in the case of samples electrodeposited in a chloride bath the measurement of a reflectance signal in the IR wavelengths range shows the presence of free electrons, and evidences the doping effect of chlorine. The values of the doping level and the optical mobility for an as-grown ZnO layer deposited in a bath containing 0.1 M of KCl, calculated with the Drude model are 1.7 × 10^20^ cm^−3^ and 12 cm^2^.V^−1^.s^−1^ respectively.

### Annealing temperature influence

We showed in a previous paper^12^ the need of a low temperature thermal post treatment to increase the electrical performances of the electrodeposited zinc oxide layer. As far as the application of front contact of CIGS solar cell is targeted the annealing temperature shall not exceed 200°C in order to prevent the degradation of the p-n junction. Thus the temperature of the post treatment has been varied from 80°C to 200°C.

The reflectance spectra as function of annealing temperature and the corresponding fit values are presented in Figure 3. The increase of process temperature leads to a shift of the plasma frequency and a slight drop of the reflectance to higher wavelengths. This trend is the mark of a decrease of the optical carrier density and an increase of the optical mobility. This assumption is confirmed by the values of N_opt_ and μ_opt_ which evolve linearly in an opposite manner with the temperature. When the annealing temperature varies from 80°C to 200°C, the carrier density ranges from 2.2 × 10^20^ cm^−3^ to 8 × 10^19^ cm^−3^ and the optical mobility from 12 to 22 cm^2^ V^−1^ s^−1^. This study shows the ambivalent effect of the annealing step which improves the intra-grain mobility but also decreases the apparent N_opt_ at higher temperatures.

### Chloride concentration influence

**Figure 4:**
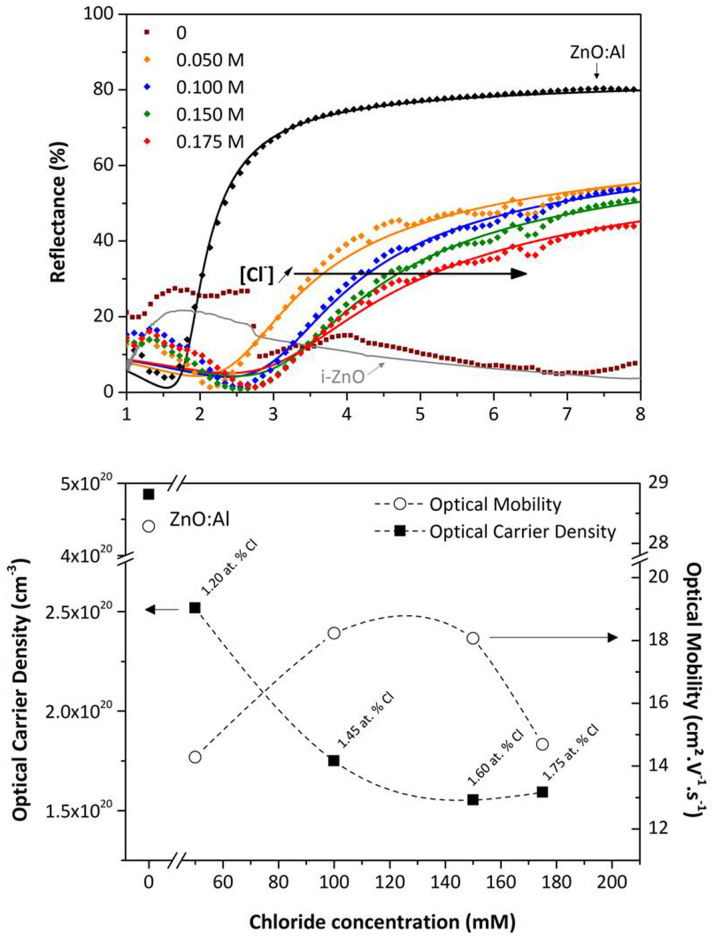
Impact of the chloride concentration on the optoelectronic properties of the electrodeposited ZnO. Top: Experimental and simulated reflectance spectra of electrodeposited ZnO films after the lift-off step, as a function of the chloride concentration compared to i-ZnO and ZnO:Al reference materials. Bottom: Optical mobility and carrier concentration extracted from fitted reflectance data. The chloride contents measured by EDX are mentioned.

The influence of chloride concentration on optical reflectance and electrical properties of electrodeposited ZnO layer has been investigated and is presented in Figure 4.

The increase of the chlorine content is accompanied by a decrease of the reflectance intensity from 50% to 40% and by a shift of the plasma frequency to higher wavelengths. At the lowest concentration, optical carrier density is the highest with 2.5 × 10^20^ cm^−3^ and decreases down to 1.5 × 10^20^ cm^−3^ for [Cl] = 0.175 M. Thus, the doping level does not linearly evolve with the chloride concentration suggesting a limitation of the chlorine doping effect. This behavior is in agreement with the observations of Lee *et al.*^23^ and with our simulation results which show that a part of chlorine atoms may fill interstitial sites and becomes inefficient as doping agent. The optical mobility presents a maximum as a function of the chloride concentration (and consequently as a function of atomic concentration of chlorine) ranging between 15 and 20 cm^2^ V^−1^ s^−1^. The first addition of chloride tends to increase the grain size and the general film quality leading to an enhancement of the free carrier mobility. But the introduction of a too high atomic concentration of chlorine in the lattice provokes a decrease of the mobility which can be related to intragrain bulk scattering. For example, 1% of chlorine in the lattice corresponds to an atomic concentration of 4.2 × 10^20^ cm^−3^. Thus according to the EDX measurement the ratio of “active chlorine” range between 45% (for [Cl] = 0.05 M) and 20% (for [Cl] = 0.175 M). The introduction of a large amount of electrically inactive impurities, through compensating defects for example, explains a mobility lower than the one obtained with aluminum doping. Bang gap evolution.

**Figure 5:**
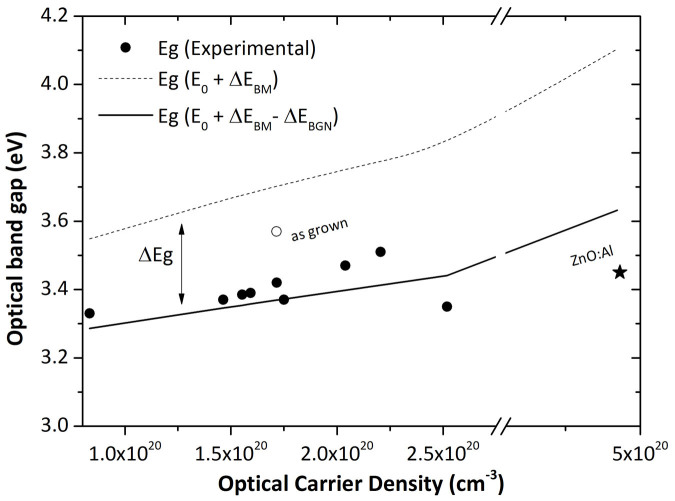
Experimental (circles) and calculated (line) optical band gap vs optical carrier density determined by reflectance fitting.

For all samples the band gap (extracted from the transmission spectra shown in the supplementary section) is superior to the intrinsic zinc oxide one which is commonly estimated at 3.3 eV^24^. The insertion of chlorine in the layer slightly increases the optical band gap. A 3.33 eV band gap is measured for the chlorine-free electrodeposited zinc oxide and rises to 3.40 eV for 0.2 M chloride concentration. This phenomenon suggests a widening of the gap in accordance with the Burstein-Moss band-filling effect^25^,^26^ related to an increase of the carrier density and described by the following relation:
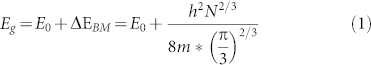
where *E_g_* is the optical band gap of the material, *E_0_* the intrinsic band gap (*E_0_* = 3.3 eV for ZnO) and *h* the Planck constant and N, the carrier concentration. Nevertheless E_g_ calculations with N_opt_ obtained from reflectance measurement are not correlated with experimental E_g_ as shown in Figure 5. An important difference can be observed between calculated (dashed line) and experimental values (circles). This phenomenon has been described by Roth *et al.*^27^,^28^. Below a critical carrier density estimated at 2 ~ 3 × 10^19^ cm^−3^ the experimental band gap matches well with the Burstein-Moss model. Above this carrier density a band gap narrowing (ΔE_BGN_) occurs, attributed to a semiconductor-metal transition, and Burstein-Moss model is no longer relevant. For heavily doped semiconductors like zinc oxide, the band gap shrinkage is proportional to N^1/3^ and can be plotted (solid line) according to the relation
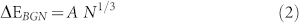
where A is an adjusted parameter. In our study the A is equal to about 6 × 10^−8^ eV cm, in good agreement with Roth^28^ and Steinhauser^29^ who respectively found a value of 3.6 × 10^−8^ eV cm for sputtered and MOCVD ZnO samples and 5.4 × 10^−8^ eV cm for LPCVD ZnO:B samples. The experimental band gap of the non annealed ZnO layer (white circle) is not correlated. The very high value of the band gap for the as-grown sample may be due to the presence of hydroxide sub-products which are transformed into oxide during the annealing process.

## Resistivity

**Figure 6:**
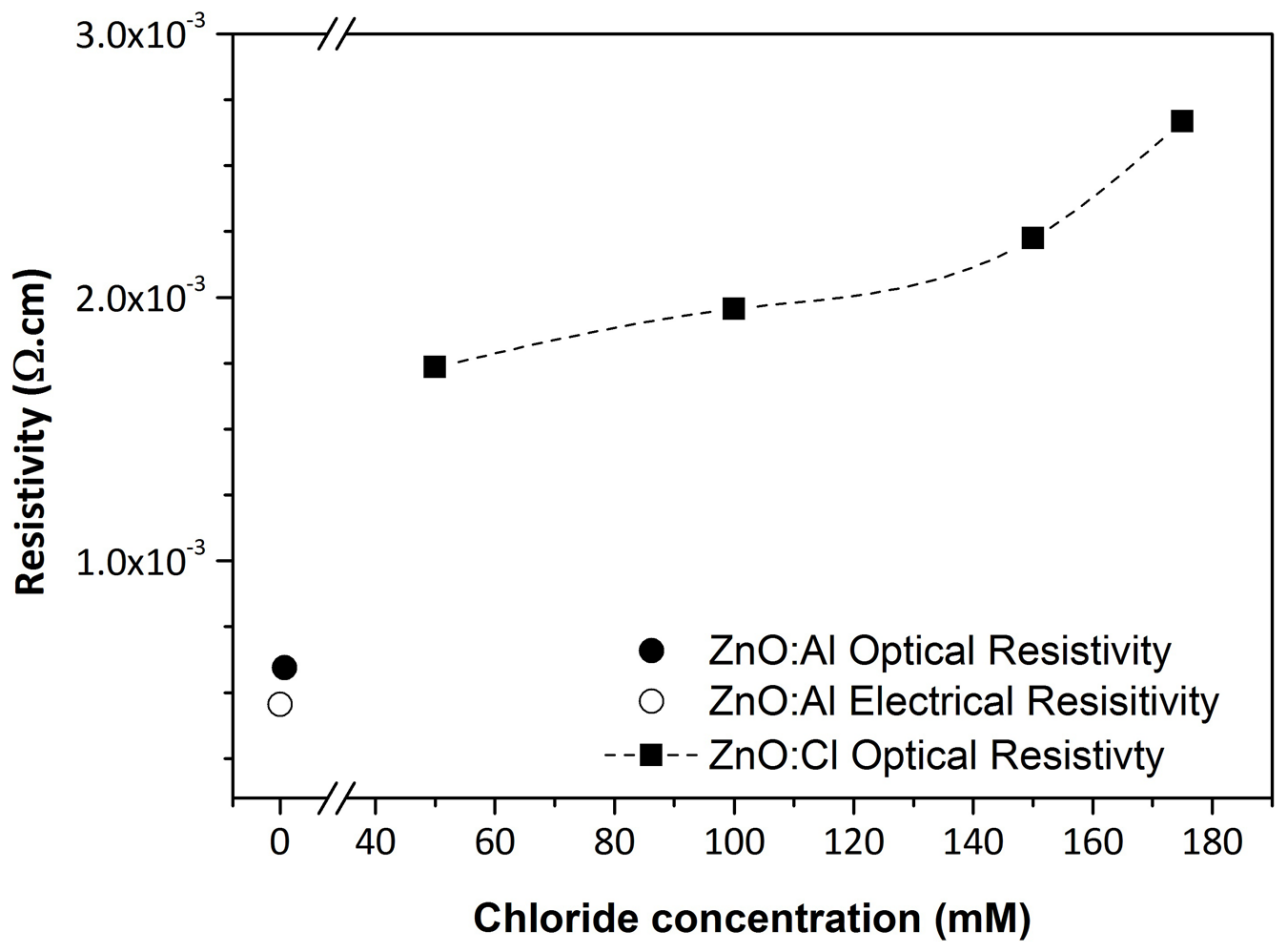
Resistivity calculated from optical data extracted from the reflectance measurement. In the case of ZnO:Al the electrical sheet resistivity is also mentioned for comparison.

The values of resistivity and sheet resistance have been calculated from the optical data (the so called optical resistivity ρ_opt_) are presented in Figure 6. They represent the maximum reachable electrical performance of the ZnO layer regardless of the morphology of the film *i.e.* grain boundaries effects. In the case of our standard sputtered ZnO:Al, ρ_opt_ and R_opt_ are calculated to be 4.7 × 10^−4^ Ω.cm and 12 Ω respectively for a layer thickness of 380 nm, these values have to be compared to sheet resistance measured by Hall effect R_□_ = 16 Ω. In general the electrical characteristics of the electrodeposited material are slightly lower than those of the sputtered one. In order to reach the same conductivity level, larger thicknesses have to be deposited. A balance has to be found between the electrical performances and the transparency of the layer. For the electrodeposited samples the best optical parameters while keeping the transparency high (ρ_opt_ = 1.9 × 10^−3^ Ω.cm and R_opt_ = 12 Ω for a thickness of 1.6 micron) have been obtained for a chlorine concentration of 0.1 M. The transmission curves obtained on ZnO:Cl layer transferred of glass/epoxy substrates are shown in the supplementary section. A further increase of the chlorine concentration tends to slightly lower the optical performances of the electrodeposited material, by mainly decreasing the intragrain mobility. But, this parameter is not the only factor to consider when optimizing a front contact layer. A dense morphology is also a prerequisite to ensure efficient long distance diffusion. A choice has to be made considering both the intragrain performances and the compactness of the film. The best cells results with a ZnO:Cl layer as window layer presented below have been obtained at [Cl] = 0.15 M.

## Device properties

**Figure 7:**
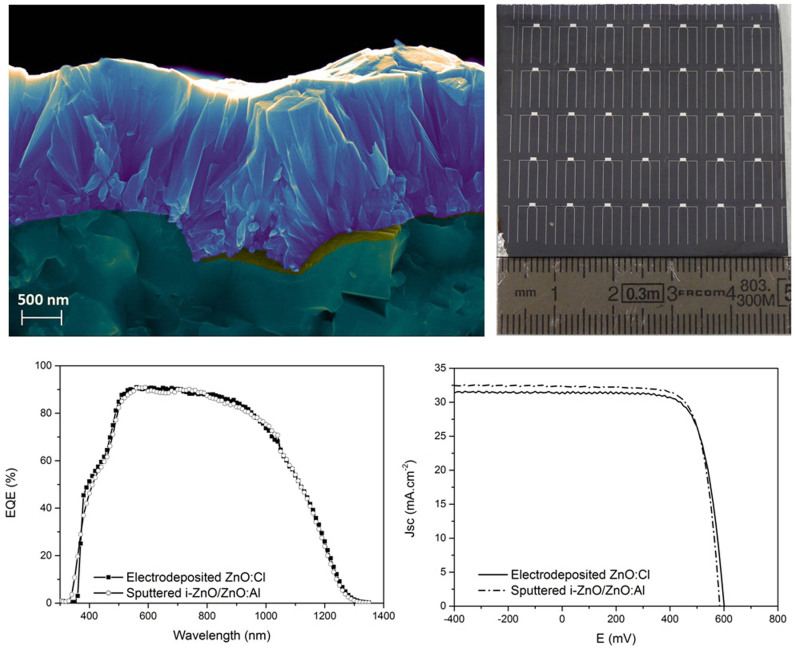
Morphology and performances of the electrodeposited ZnO as front contact of a CIGS solar cell. SEM image of a cross section of an electrodeposited ZnO on a CIGS(electrodeposited)/CdS substrate. Image of a 5 × 5 cm^2^ sample divided into 0.5 cm^2^ cells. I-V curve and spectral response of 0.5 cm^2^ solar cells based on electrodeposited CIGS and terminated by an electrodeposited single layer of ZnO:Cl and a sputtered bi-layer of i-ZnO/ZnO:Al.

Electrodeposition or chemical growth of ZnO as front contact for CIGS devices has already been realized with success. Nevertheless up to now, the substrate used to perform the chemical or electrochemical growth was constituted of the stack Mo/CIGS/CdS/i-ZnO where i-ZnO was synthesized by magnetron sputtering^13^,^14^ or spin coating process^30^. In the present work and at contrary to previous studies, the window layer deposition directly occurs on CdS buffer layer. The growth has been carried out under illumination. The p-n junction formed by CIGS and CdS shows a blocking behavior at the potential applied during the electrodeposition, and then the deposition has to be photo-assisted. The photocurrent produced under illumination is consumed by the electrochemical reaction. These process allows the deposition of homogenous TCO films on large substrates (up to 15 × 15 cm^2^ area samples have been tested). In this work 0.5 cm^2^ cells defined by mechanical scribing on a 5 × 5 cm^2^ sample and terminated by a metallic contact as presented in figure 7, have been measured. No additional antireflection coating was applied. A SEM cross section of the ZnO:Cl grown on a electrodeposited CIGS/CdS shows that the electrochemical layer is dense, compact and covering. In order to compensate the higher resistivity of the ZnO:Cl thicker films are grown (1600 nm) compared to sputtered ZnO:Al front contact (380 nm).

The external quantum efficiencies (Figure 7) and current-voltage curves obtained on the best cells with both tested ZnO layers coincide. The record and average efficiencies (Table 1) measured with an electrodeposited front contact are slightly lower than those obtained with a sputtered one. The higher fill factor measured with ZnO:Al is correlated with its lower resistivity, but unexpectedly and despite the absence of intrinsic ZnO, the cells terminated with an electrodeposited layer show the best open circuit potential. As far as we know, an efficiency of 14.3% is reported for the first time for solar cell made only from atmospheric processes (excepted for the back contact molybdenum layer deposition). Thus the electrodeposition appears as a credible alternative to the sputtering process to produce high performance TCO.

## Conclusion

We presented in this paper a theoretical and optical study of ZnO layers deposited by electrochemical means. Optimization of the deposition conditions and the post treatment temperature allow one to obtain electrical and optical properties close to the ones obtained for a sputtered TCO. The role of the chlorine as doping element has been elucidated by ab-initio simulations and optical measurements. A lift-off technique was implemented to prepare electrodeposited ZnO:Cl layers for full optical characterization. The electrodeposition is shown to be a powerful tool to produce highly transparent and conductive ZnO. The high material quality is confirmed by the performances of the cells terminated with an electrodeposited front contact. The efficiencies obtained with a cell terminated by a single electrodeposited ZnO layer are comparable to those measured terminated with the classical sputtered bi-layer i-ZnO/ZnO:Al. A cell only produced by atmospheric processes (from CIGS to ZnO) and reaching the very encouraging efficiency of 14% is also reported in this paper. This result opens the perspective to an all atmospheric and low cost production line of CIGS based solar cells. Experiments are in progress to reproduce these results on larger area (30 × 60 cm^2^) and test the long term stability of electrodeposited ZnO:Cl material.

## Experimental details

The electrodeposition is performed at a low temperature fixed at 80°C in a solution containing zinc chloride in a concentration of 5 × 10^−3^ M. The solution is saturated by bubbling oxygen gas used as oxygen precursor^5^. For the study of the impact of a low temperature post treatment, the depositions are performed in a bath with a chloride concentration set at 0.1 M. The annealing temperature varied between 80°C to 200°C.

In order to emphasize the effect of the chloride content, a variable concentration of potassium chloride ranging from 0.05 to 0.2 M is introduced in the electrolyte. The ZnO layer is grown on molybdenum coated glass substrates. For the sample deposited without chloride, deposition takes place in the same conditions but in zinc nitrate electrolyte at 0.05 M. In this case, nitrate ions are used as oxygen precursor^6^. In order to stabilize the electrical properties of the films the samples are annealed at 150°C during 30 minutes.

Two sputtered films have been also grown on glass by sputtering as reference materials, one is highly extrinsically aluminum doped and the second is not intentionally doped. These films which are referred as ZnO:Al and i-ZnO (for intrinsic ZnO) are 376 nm and 200 nm respectively.

The deposition on CdS/CIGS substrate is performed without any seed layer under illumination^13^. The ZnO growth is followed by an annealing at 150°C during 30 minutes.

To complete the solar cell based on electrodeposited CIGS, the electrodeposited ZnO have been grown on 15 × 15 cm^2^ samples, extracted from 60 × 120 cm^2^ substrates produced in NEXCIS. For characterisation needs these samples have been cut into 5 × 5 cm^2^ and finally 0.5 cm^2^ cells have been tested. Before electrical measurements a Ni/Al metallic grid has been evaporated on the ZnO surface.

For the reference cell, a bilayer i-ZnO (50 nm)/ZnO:Al (370 nm) is grown on the top of the classic CIGS/CdS stack.

## Author Contributions

F.T. and J.R. contributed equally to this work and wrote the manuscript. F.T., A.V. and J.C. electrodeposited the ZnO layer and characterized the complete cells. J.V. realized the ab-initio calculation study and wrote the simulation section of the manuscript. S.C. carried out the reflection measurements and L.L. fitted and analyzed the data. M.P. designed the metallic contact. S.B. carried out the SEM characterization. C.B., S.J. and P.P.G. built and provided the Glass/Mo/CIGS/CdS stack. J.R. and D.L. have supervised the work. All authors reviewed the manuscript.

## Supplementary Material

Supplementary Information Supplementary materials

## Figures and Tables

**Table 1:** Comparison between the maximum and average performances of 0.5 cm^2^ solar cells (based on electrodeposited CIGS) terminated by an electrodeposited single layer of ZnO:Cl and a sputtered bi-layer of i-ZnO/ZnO:Al

	Eff (%)	Voc (mV)	Jsc (mA.cm^−2^)	FF (%)
CIGS (ED)/CdS/i-ZnO (Sp)/ZnO:Al (Sp) Max	14.9	605	33.3	73.8
CIGS (ED)/CdS/i-ZnO (Sp)/ZnO:Al (Sp) Average	14.4	600	33.4	72
CIGS (ED)/CdS/ZnO:Cl (ED) Max	14.3	621	33.8	68
CIGS (ED)/CdS/ZnO:Cl (ED) Average	13.8	616	33.8	66.5
