# Supplementary material for: Electrodeposition of ZnO window layer for an all-atmospheric fabrication process of chalcogenide solar cell
Source: Sci Rep. 2015 Mar 10;5:8961. doi: 10.1038/srep08961 (PMC4353998; doi:10.1038/srep08961)
Supplement: Supplementary Information — Supplementary materials [file srep08961-s1.docx]

Supplementary materials

**Electrodeposition of ZnO window layer for an all-atmospheric fabrication process of chalcogenide solar cell**

Fabien Tsin^1^, Amélie Venerosy^1^, Julien Vidal^1^, Stéphane Collin^2^, Johnny Clatot^1^, Laurent Lombez^1^, Myriam Paire^1^, Stephan Borensztajn^1^, Cédric Broussillou^3^, Pierre Philippe Grand^3^, Salvador Jaime^3^, Daniel Lincot^1^, Jean Rousset^1^*.

1. IRDEP, Institute of Research and Development on Photovoltaic Energy, UMR 7174 CNRS EDF Chimie ParisTech, 78401 Chatou, France
2. LPN, Laboratoire de Photonique et de nanostructures (LPN-CNRS), Route de Nozay, 91460 Marcoussis, France
3. NEXCIS Photovoltaic Technology, 13790 Rousset, France

Corresponding author: [jean.rousset@edf.fr](mailto:jean.rousset@edf.fr)

**Experimental details**

Film deposition and lift-off process

Electrochemical depositions have been performed in a transparent quartz reactor with three-electrode configuration, using a saturated calomel electrode (SCE +0.248 V vs. NHE) as reference electrode, a Zn foil as counter-electrode and a conductive or semiconductor substrate as working electrode. This last one can be a molybdenum coated glass substrate or a CdS coated CIGS substrate. Deposition experiments have been performed under control potential (-1.0 V vs. SCE) and under illumination in the case of electrodeposition on CdS coated CIGS substrate. The thickness of the films is controlled by the charge consumed during the growth and ranges from 1 micron to 1.6 micron.

Conductive substrates needed for the electrochemical growth are not suitable to carry out accurate optoelectronic characterizations due to electrical and optical interactions with the ZnO layer. Thus, we developed a lift-off technique to transfer the ZnO layer from its growth substrate to a host material for characterization. The film of zinc oxide is taken off its substrate by applying epoxy resin between the film and a glass lamella. A mechanical pressure allows taking off the ZnO layer and obtaining samples composed of the following stack: glass/epoxy/ZnO. This technique is ideal for optical characterizations because the epoxy resin is transparent, inert and amorphous.

Characterization

The morphology and composition of zinc oxide layer are determined by scanning electron microscope (Zeiss-Merlin compact) coupled with an Electron dispersive X-Ray Spectrometer (EDS).

Direct electrical measurements for the determination of the doping level and the carrier mobility such as Hall effect are difficult due to microcracks appearance in the layer during the lift-off. In this paper the insertion of chloride and its doping effect is evaluated by reflectance measurement in the Infrared. Reflection spectra were measured with a Fourier-transformed infrared spectrometer (Bruker Vertex 70) and a MCT detector. A gold mirror was used as a reference.

The transmittance spectra were measured between 250 and 2000 nm by using a Perkin Elmer lambda 900 spectrophotometer for band gap evaluation.

**Theoretical background**

Ab initio Simulation

Ab initio calculations were performed using the VASP package [1] within the PAW framework [2,3]. The GGA+U functional with U=6 eV for Zn 3d orbitals were employed throughout the study. A Γ-centered 4x4x4 k-point sampling and 480 eV kinetic energy cutoff were necessary to converge total energy of a 72-atom supercell of ZnO. Image charge, band filling and finite size corrections were applied together with band gap correction calculated from GW [4, 5]. The good agreement between the transition level ε(0/2+) of V_O_ calculated in this study and the one calculated using more advanced technique validate the corrective approach used in this study [6].Within the pH and temperature range employed in this study, ZnO will precipitate preferentially with respect to Zn(OH)_2_ and therefore chemical potential of Zn and O are related by the simple relation Δμ_Zn_+Δμ_O_=ΔH^0^_f_(ZnO). The FERE method [7] was used to compute the formation enthalpies ΔH^0^_f_(T=298K) for ZnO, ZnCl_2_ and binary oxides and halides and the fitted elemental chemical potential were subsequently used as references. The inclusion of a dopant A is limited by the formation of Zn-associated binary compound Zn_x_A_y_. Therefore, chemical potential of species A is expressed as

$\Delta\mu_{A}=\frac{1}{y}\times\left[ \Delta H_{f}^{0}\left( {Zn}_{y}A_{y} \right)-x\Delta\mu_{Zn} \right]$ (1)

Drude Model

The optical behavior of electrodeposited zinc oxide can be described by the Drude model applied to doped semiconductors [7, 8]. In this case, frequency dependant dielectric function *ε_r_* can be expressed as

$\varepsilon_{r}\left( \omega\right)=\varepsilon_{opt}\left( 1-\frac{\omega_{p}^{2}}{\left( \omega^{2}+i\Gamma\omega\right)} \right)$ (2)

where *ε_opt_* is the value measured in the transparent spectral region below the interband absorption edge and is known from the refractive index of the undoped semiconductor, *ω_p_* is the plasma frequency and *Γ* is a damping frequency which can be respectively expressed by the equation (3) and (4)

$\omega_{p}^{2}=\frac{N_{opt} e^{2}}{\varepsilon_{opt} \varepsilon_{0}m^{*}}$ (3)

$\Gamma=\frac{e}{\mu_{opt}m^{*}}$ (4)

where N_opt_ is the free carrier density, μ_opt_ the optical mobility, e the electron charge, ε_0_ the vacuum permittivity and m* the electron effective mass – assuming m*= 0.28m_e_ where m_e_ is the electron mass. Moreover the reflectance of the material can be written as the function of the dielectric function as follows (5):

$R=\frac{\left( \sqrt{\varepsilon}-1 \right)^{2}}{\left( \sqrt{\varepsilon}+1 \right)^{2}}$ (5)

From these expressions, it is clear that the optical carrier density and the optical mobility can be obtained from the fit of experimental reflectance spectra.

**Composition aspect**

The figure 3 shows the insertion of chlorine atoms in the ZnO layer as a function of the chloride concentration dissolved in the electrolyte.

**Transmittance study**

Figure 4 shows the transmittance spectra of the ZnO:Cl/epoxy/glass structure and the optical band gap (extracted from the transmittance spectra) evolution as a function of chloride concentration and annealing temperature. The spectra of sputtered i-ZnO and ZnO:Al reference layers are also presented. The first one, very lightly and unintentionally doped, is highly transparent in the visible and infrared range with a transmittance equal to 80%. The second one, highly doped, shows a high absorption in the near infrared from 1000 to 2000 nm.

Chloride concentration effect

The transmittance spectra of the sample synthesized in a nitrate electrolyte is similar to the one of intrinsic zinc oxide, which confirms its weak doping level. In general and despite a larger thickness (ranging from 1 micron to 1.6 micron) in comparison with that of the sputtered film (380 nm), the electrodeposited ZnO films show an increased transparency in the range of the solar spectrum and more specifically in the NIR. This effect is particularly visible in the case of the layer grown in the chlorine most concentrated bath. Indeed the transparency of the electrodeposited ZnO:Cl layer slightly increases in the visible range as chloride concentration rises. The evolution of the film morphology and particularly its compactness could be the cause of this increase. Indeed the pen-shaped morphology obtained at low chloride concentration –and more generally a rough film- could lead to multiple reflections of the incident light and limit the transmission at shorter wavelengths. In the near infrared range, transmittance of electrodeposited samples is higher than the sputtered Al-doped sample but lower than the intrinsic one. This phenomenon is related to the free carrier absorption which increases with the doping level.

Annealing temperature impact

Each sample, after annealing at temperatures ranging from 80°C to 200°C, is found to be highly transparent with a transmittance superior to 80% in the visible range (Figure 4). The transparency appears to be independent on annealing temperature in this wavelength range. On the contrary, transmittance intensity increases in the near infrared range as the annealing temperature rises. This phenomenon has been already described in a previous paper [9] and has been related to the decrease of the free carrier concentration demonstrated by reflection measurements. Moreover a clear impact of the annealing can be observed in the near-ultraviolet range with a shift of the absorption edge to higher wavelengths as the annealing temperature rises. The temperature increase leads to a shift of the optical band gap to lower energies. The optical band gap ranges from 3.57 eV, for as-grown sample, to 3.33 eV for annealed sample at 200°C.

**Test of complete cell**

In the case of the coevaporated CIGS, very small sized cells are tested (0.1 cm^2^). For this cell geometry the best result have been obtained with a 0.8 micron thick electrodeposited ZnO:Cl. At this thickness, electrodeposited ZnO shows a better transparency than the standard i-ZnO/ZnO:Al (50 nm/ 370 nm) sputtered bi-layer. This improved transmission impacts positively the quantum efficiency for wavelengths superior to 700 nm. As a consequence, the short circuit current is significantly higher than for the sputtered ZnO film.

In a non controlled manner, we also observed, a slight increase of the quantum efficiency (Figure 5) in the ultraviolet range. This is probably due to the dissolution of a part of the CdS caused by our deposition conditions [10] and particularly the presence of oxygen in the bath and/or illumination.

1. Lany, S. & Zunger, A. Assessment of correction methods for the band-gap problem and for finite-size effects in super cell defect calculations: Case studies for ZnO and GaAs. *Phys. Rev. B* **78,** 235104 (2008).
2. Kresse, G. & Joubert, D. From ultrasoft pseudopotentials to the projector augmented-wave method. *Phys. Rev. B* **59,** 1758- (1999).
3. Kresse. G & Furthmüller, J. Efficient iterative schemes for ab initio total-energy calculations using a plane-wave basis set. *Phys. Rev. B* **54**, 11169 (1996).
4. Peng, H., Scanlon, D.O., Stevanovic, V., Vidal, J., Watson, G.W. & Lany, S. Convergence of density and hybrid functional defect calculations for compound semiconductors. *Phys. Rev. B* **88,** 115201 (2013).
5. Lany, S & Zunger, A. Many-body calculation of the oxygen vacancy in ZnO. *Phys. Rev. B* **81,** 113201 (2010).
6. Stevanovic, V., Lany, S. Zhang, X. & Zunger A. Correcting density functional theory for accurate predictions of compound enthalpies of formation: Fitted elemental-phase reference energies. *Phys. Rev. B* **85,** 115104 (2012).
7. Fox, M. *Optical Properties of Solids* (Oxford University Press, 2001).
8. Jin, Z.-C., Hamberg, T. & Granqvist, C.G. Optical properties of sputter-deposited ZnO:Al thin films. *J. Appl. Phys.* **64,** 5117-5131 (1988).
9. Rousset, J., Saucedo, E., Herz, K. & Lincot, D. High efficiency CIGS based solar cells with electrodeposited ZnO:Cl as transparent conducting oxide front contact. *Prog. Photovolt.: Res. Appl.* **19,** 537-546 (2011).
10. Meissner, D., Benndorf, C. & Memming, R. Photocorrosion of cadmium sulfide: Analysis by photoelectron spectroscopy. *Applied Surface Science* **27,** 423-436 (1987).


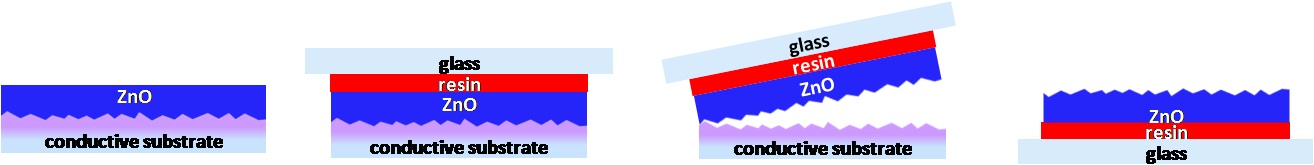


Figure 1. Schematic description of the lift off process.


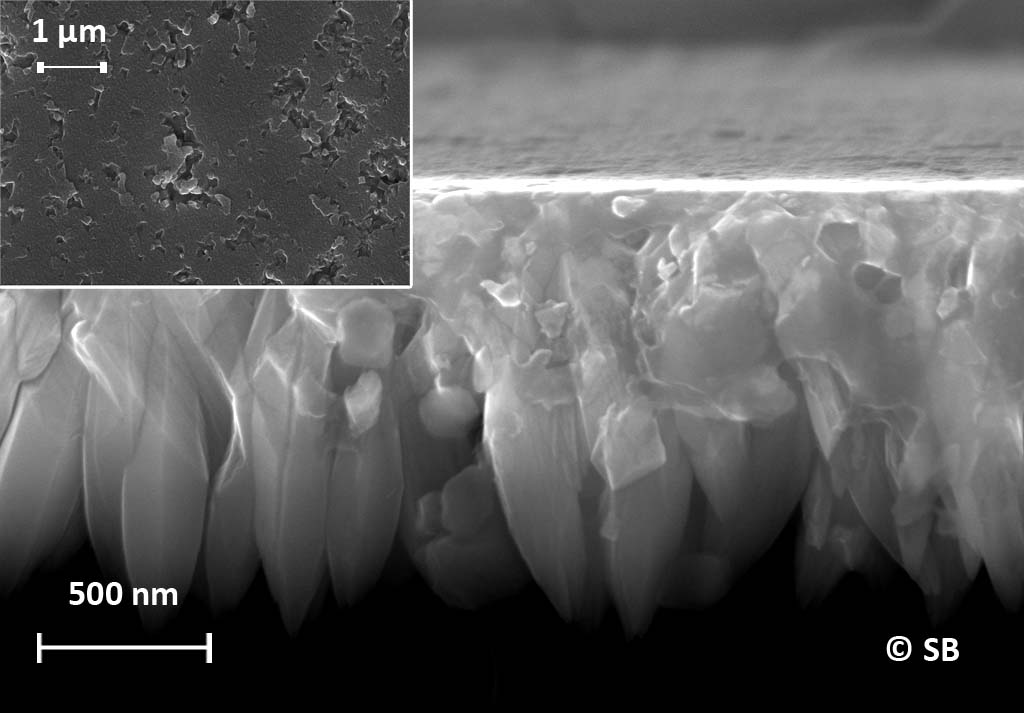


Figure 2. Layer deposited at 0.05 M and transferred on a glass/epoxy substrate


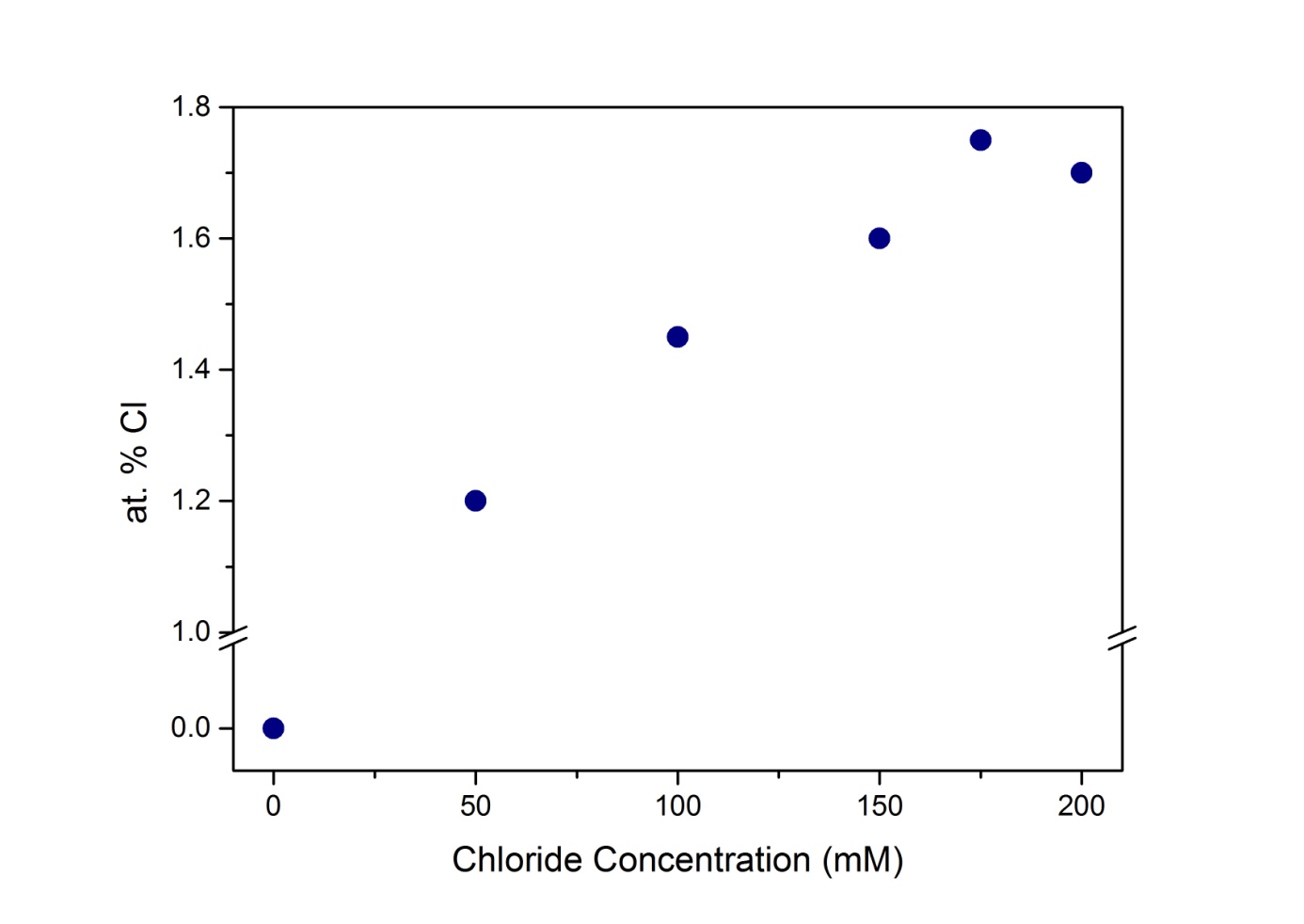


Figure 3: Electron dispersive spectrometry (EDS) measurements of the chlorine concentration in the ZnO layer as a function of the chloride concentration for [Cl^-^] ranging from 0 M to 0.2 M


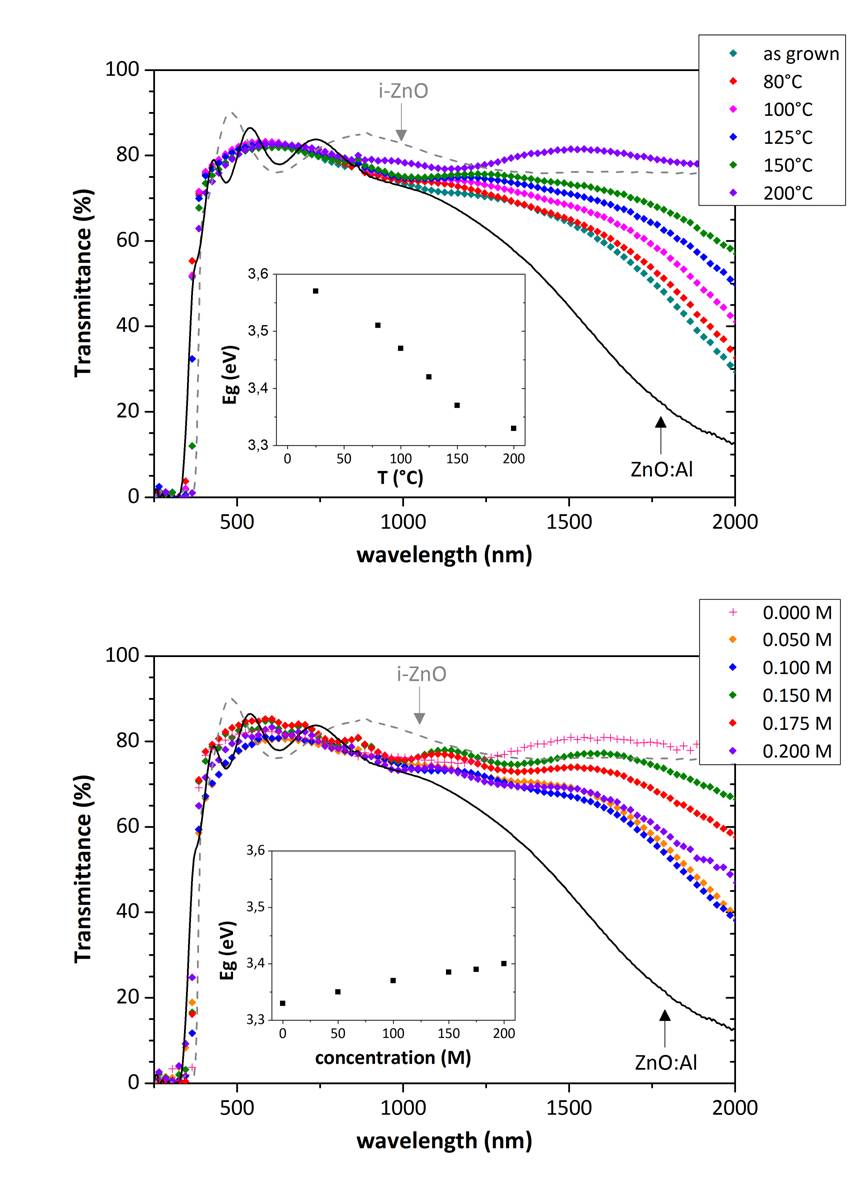


Figure 4. Optical transmittance spectra obtained from electrodeposited ZnO films after the lift-off step as function of the annealing temperature (top) end the chloride concentration (bottom) compared to sputtered i-ZnO and ZnO:Al data. Insert: Band gap values extracted from the transmittance spectra.


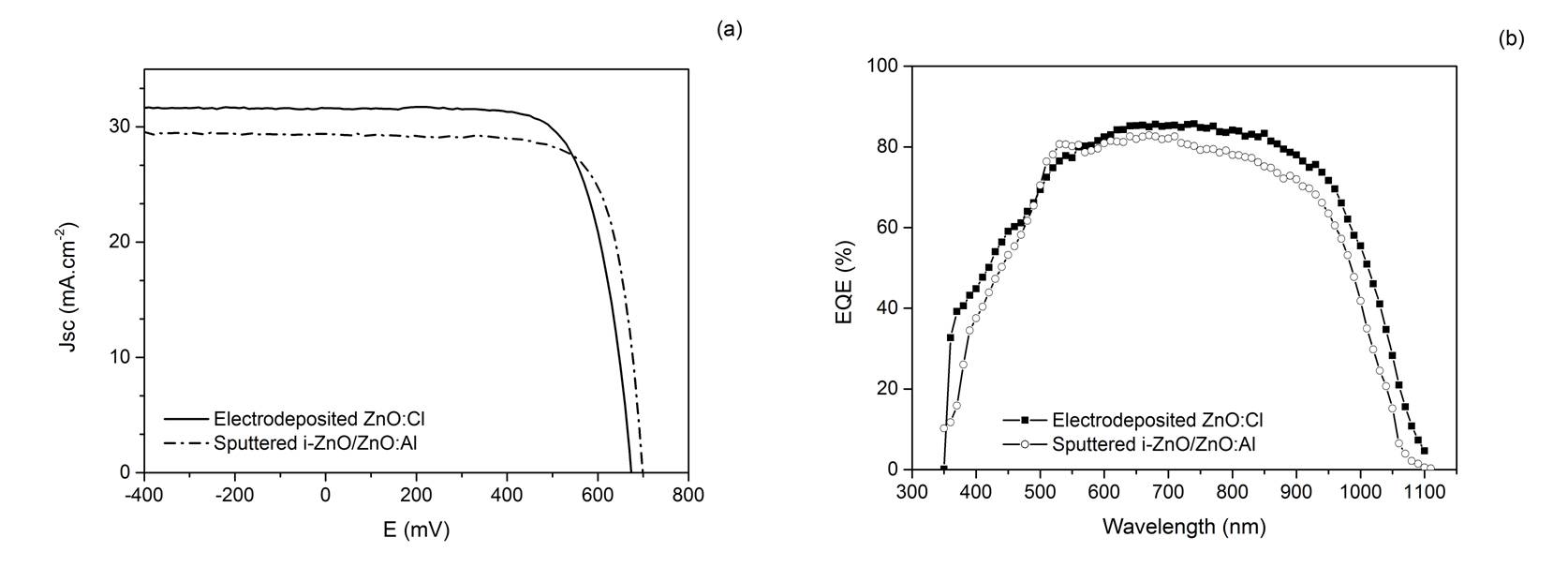


Figure 5. I-V curve (a) and spectral response (b) of 0.1 cm² solar cells based on sputtered CIGS terminated by an a sputtered bi-layer of i-ZnO/ZnO:Al

|  | Eff  (%) | Voc  (mV) | Jsc  (mA.cm^-2^) | Jsc EQE  (mA.cm^-2^) | FF (%) |
| --- | --- | --- | --- | --- | --- |
| CIGS (Sp)/CdS/i-ZnO (Sp)/ZnO:Al (Sp) | 15.2 | 700 | 29.4 | 28.2 | 74 |
| CIGS (Sp)/CdS/ZnO:Cl (ED) | 15.1 | 673 | 31.6 | 30.2 | 70 |

Table 1. Comparison between the maximum and average performances of 0.1 cm² solar cells (based on electrodeposited CIGS) terminated by an electrodeposited single layer of ZnO:Cl and a sputtered bi-layer of i-ZnO/ZnO:Al
